# Hypogammaglobulinemia Observed One Year after Rituximab Treatment for Idiopathic Thrombocytopenic Purpura

**DOI:** 10.1155/2018/2096186

**Published:** 2018-03-21

**Authors:** Bilal Ahmad Shoukat, Osama Ali, Dileep Kumar, Muhammad Bilal Gilani, Adeela Zahid, Shaheer Aslam Joiya, Maqsood Anwar Malik

**Affiliations:** ^1^Our Lady's Hospital, Navan, Meath, Ireland; ^2^Providence Hospital, Washington, DC, USA

## Abstract

We present the case of a 19-year-old female with severe hypogammaglobulinemia after having had treatment with rituximab for idiopathic thrombocytopenic purpura requiring intravenous immunoglobulins. She was admitted with the diagnosis of left-sided pneumonia with parapneumonic effusion. The patient was treated with piperacillin/tazobactam after having a poor response to co-amoxiclav. The patient had been tested for immunoglobulin levels, and the levels were very low. She has a history of ITP for which she received steroids. She also received rituximab for the same on four separate occasions, and the last one was about 1 year ago.

## 1. Background

Humoral immunity is dependent on a full repertoire of mature B-lymphocytes capable of adequately mounting a primary and secondary immune response. If the abovementioned phenomenon is impaired, it will invariably lead to severe bacterial infections as can be evaluated by the disease spectrum in inborn and acquired immunodeficiencies. Regarding the former, common variable immune deficiency (CVID) is a heterogeneous entity characterized by varying degrees of hypogammaglobulinemia and recurrent bacterial infections [1, 2].

Rituximab, a chimeric monoclonal antibody binding CD20, is increasingly used in the treatment of B-cell lymphomas [3, 4]. Rituximab is responsible for causing a rapid depletion of CD20-expressing B-cell precursors and mature B-cells, which remain at very low or undetectable levels for months before returning to pretreatment levels [5]. Rituximab can lead to a state of immunosuppression through B-cell depletion and also through the development of late-onset neutropenia and hypogammaglobulinemia [6, 7]. Although rituximab is known to cause hypogammaglobulinemia in people with normal immunoglobulins prior to treatment, its effect on immunoglobulins is transient [8]. Here, we describe an interesting case of a young female who developed severe hypogammaglobulinemia diagnosed 1 year after receiving rituximab requiring the administration of immunoglobulin therapy (as noted in one study in up to 4.2% of 243 patients [9]) [10].

## 2. Case Presentation

A 19-year-old female presented to the emergency department with chest pain, shortness of breath, and productive cough for a few weeks. Her past medical history included immune thrombocytopenic purpura, initially treated with tapering dose of steroids followed by a course of rituximab after the poor response to steroid treatment. She had received four cycles of rituximab, last one approximately a year ago. We did not find any record of her immunoglobulin level being checked before and after the treatment. Physical examination showed left-sided chest crackles on auscultation and lymphadenopathy in the cervical, axillary, and inguinal regions. Initial investigations revealed low hemoglobin, high C-reactive protein, and low globulin levels. Her chest X-ray showed left-sided pleural effusion and basal consolidation. A provisional diagnosis of pneumonia with parapneumonic effusion was made, and she was treated with piperacillin/tazobactam after poor response to co-amoxiclav plus clarithromycin. Due to poor response to antibiotics and generalized lymphadenopathy, we decided to perform CT-neck, thorax, abdomen, and pelvis to lookout for possible hematological malignancies. CT revealed enlarged lymph nodes in cervical, mediastinal, bilateral axillary, periaortic, and mesenteric regions and left-sided pleural effusion. There was a possibility of lymphoma. She had a pleural fluid drainage, and it was then studied for microscopy and histopathology. Pleural fluid histopathology showed no evidence of malignancy. Lymph node biopsy was done to exclude malignancy. Her immunoglobulin concentration was determined, and there was complete absence of IgM, IgA, and IgG. The immunologist suggested commencing the patient with immunoglobulin replacement therapy urgently, and the case was diagnosed as having severe hypogammaglobulinemia most likely secondary to rituximab therapy. Her status improved considerably following her acute admission to the hospital. We did not find any record of immunoglobulin levels prior to this admission.

## 3. Investigations

On investigation, the TSH level was 1.30 IU/L (0.35–4.94). Vitamin B12 level was 279 pg/ml (189–1162), folate 10.7 ng/ml (3.1–2.0), ferritin 72 ng/ml (5–204), globulin level 15 g/l (21–36), urea 1.9 mmol/l (2.5–6.7), creatinine 52 *µ*mol/l (50–98), albumin 36 g/l (35–50), total protein 51 g/l (60–80), hemoglobin 10.7 g/dl (12.0–15.0), white blood cell count 6.6 × 10^9^/L (4.0–10), neutrophil count 4.96 (2.0–7.0), lymphocyte count 1.05 (1.0–3.0), monocyte count 0.47 (0.2–1.0), eosinophil count 0.07 (0.1–1.0), basophil count 0.02 (0.02–0.1), and platelet count 224 × 10^9^/L (150–410).

Blood film showed anisocytosis and small number of pencil cells.

Pleural fluid microscopy revealed RBC count of 1700 cmm and white blood cell count of 600 cmm. However, no bacteria were seen on Gram staining of pleural fluid, and differentials showed polymorphs 84% and monocytes 16%. There was no growth on blood culture. Throat swab was negative for influenza virus, parainfluenza virus, adenovirus, human metapneumovirus, and respiratory syncytial virus. Lymph node biopsy histopathology reported atrophic B zones and expansion of T-cell zone and large number of CD30+ cells. Immunoglobulin concentration showed IgG 0.78 g/l (6.26–14.96), IgA 0.05 g/l (0.62–2.90), IgM 0.05 g/l (0.47–1.82), and β2 microglobulin 2.41 mg/l (1.20–2.50). The immunoglobulin levels were not repeated before commencing the intravenous immunoglobulin therapy.

The identification of mutations of ICOS, CD19, CD20, CD21, CD80, TACI, and BAFFR may be required to establish the diagnosis of CVID in this patient. But, the aim of this case report is to alert the clinicians to consider monitoring immunoglobulin levels before, during, and after treating an autoimmune condition with rituximab [9].

## 4. Differential Diagnosis

Differential diagnosis included (a) rituximab-induced hypogammaglobulinemia, (b) unmasking of CVID by rituximab, and (c) lymphoma.

## 5. Treatment

Consultant immunologist suggested commencing the patient with 500 mg/kg of IV Ig once followed by 200 mg/kg every 3 weeks.

## 6. Outcome and Follow-Up

She is currently receiving immunoglobulin replacement therapy. As per the recent correspondence received from her consultant immunologist and general practitioner, she did not have any further significant infection. She is currently under close follow-up of consultant immunologist.

## 7. Discussion

Iatrogenic hypogammaglobulinemia is an acquired hypogammaglobulinemia caused by various drugs including antirheumatic and antiepileptic medications, but clinically, it may be difficult to distinguish it from CVID. In contrast to this, common variable immune deficiency is a congenital disorder that involves low levels of most or all of immunoglobulins, lack of B-lymphocytes or plasma cells that are capable of producing antibodies, and repeated bacterial infections [11]. CVID has various clinical presentations and types of deficiencies. Deficiency of IgG and IgA is characteristic and approximately 50% of patients with CVID are deficient in IgM levels. As per the evidence, 20% of common variable immune deficiency patients develop some autoimmune diseases [12].

It is difficult to differentiate whether the hypogammaglobulinemia is caused by rituximab or the preexisting CVID has been aggravated by rituximab treatment [13], especially if there are no previous immunology and biochemistry tests available as in our case. According to the European Society for Immunodeficiencies Registry 2014, CVID has bimodal age of manifestation with peak in diagnosis between the ages of 5 and 10 years, a trough around the age of 20, and then another peak between 30 and 40 years [14]. The mean age at the onset of symptoms was 26.3 years. Therefore, it can be argued that preexisting CVID has been unmasked at this age in this patient. This patient reported no previous history of any significant infection prior to rituximab therapy. Although one episode of infection is not enough to consider the diagnosis of immunodeficiency, in this case, severity of infection, poor response to antibiotic, and a previous history of rituximab therapy led to our speculation of an acquired hypogammaglobulinemia. There are some studies that reported that patients already diagnosed with CVID and treated with rituximab showed decline in the levels of IgG. These patients were not on IgG replacement therapy [15].

Another possibility in this case can be speculated that her immune thrombocytopenic purpura could be an autoimmune manifestation of the underlying common variable immune deficiency syndrome. However, the point should be noted that only 20% of individuals with CVID develop other autoimmune conditions as stated earlier in the discussion [12]. Therefore, rituximab treatment is indeed a possible explanation for the observed hypogammaglobulinemia.

We would like to draw a reference from a study published by Patel et al., which outlines the outcomes of patients 5 years after rituximab therapy in children and adults with ITP. This extensive long-term follow-up study reports that “treatments of patients with chronic ITP which provide a curative effect without untoward toxicity or poor tolerability are highly desirable. They allow a patient to avoid the disadvantages of low platelet counts including continued platelet count monitoring, continued treatment, and possibly bleeding and/or fatigue. The use of rituximab has been an exciting development in ITP because patients may achieve complete responses lasting at least 1 year without additional treatment. However, robust data on the long-term response to rituximab are lacking for both children and adults.” [16].

## 8. Learning Points

- Lack of measurements of previous immunoglobulin levels, mutations in the gene encoding ICOS, TACI, CD19, CD20, CD21, CD80, and BAFFR, and functional and IgG subclasses make it impossible to conclude definitely that severe hypogammaglobulinemia in this is a direct effect of rituximab treatment or is in fact an underlying CVID unmasked by rituximab.
- Iatrogenic hypogammaglobulinemia can be severe enough requiring intravenous immunoglobulin therapy.
- We recommend that patients who are on treatment for immune thrombocytopenic purpura or other autoimmune conditions with rituximab should be tested for immunoglobulins at baseline level before the treatment as well as looking for any sign of immunodeficiency before, during, and after the treatment.
